# Ethanol Exposure Up-Regulates PD-L1/PD-1 Immune Checkpoint Pathway and Promotes Mammary Tumor Development

**DOI:** 10.3389/fonc.2022.874156

**Published:** 2022-06-08

**Authors:** Wenhua Xu, Linqing Wu, Mei Xu, Jia Luo, Gang Chen

**Affiliations:** ^1^ Department Pharmacology and Nutritional Sciences, University of Kentucky College of Medicine, Lexington, KY, United States; ^2^ Department of Neurology, The First Affiliated Hospital of University of Science and Technology of China, Hefei, China; ^3^ Department of Immunology, School of Basic Medical Sciences, Fujian Medical University, Fuzhou, China; ^4^ Department of Pathology, University of Iowa Carver College of Medicine, Iowa City, IA, United States

**Keywords:** alcohol, ethanol, breast cancer, immune checkpoint, T cell, PD-1, PD-L1

## Abstract

Alcohol consumption in women enhances breast cancer incidence and ethanol is the main causal factor. Compromised host immunity through immunosuppression facilitates the development of many types of cancer, including breast cancer. Immune cells in breast tissues, particularly tumor-infiltrating CD8 cytotoxic T cells, play a critical role in the host anti-tumor immunity against breast tumorigenesis. These cytotoxic T cells are the major immune cells to carry out anti-tumor immunity through their cytotoxic effector function, which can be regulated by immune checkpoint pathways. The PD-1/PD-L1 pathway (the interaction between programmed death-1, PD-1, and its ligand, programmed death-ligand 1, PD-L1) is the best characterized one. However, the effects of ethanol exposure on T cell anti-tumor immunity and how that may contribute to ethanol-enhanced mammary tumorigenicity remain unknown. FVB.Cg-Tg(Wnt1)1Hev/J transgenic mice develop spontaneous mammary tumors starting around the age of 2-3 months and have been a widely-used mouse model for breast cancer research. Using this mouse model, the current study determined the effects of ethanol on the PD-L1/PD-1 pathway and how that may contribute to mammary tumorigenesis. The results indicated that ethanol exposure enhanced mammary tumor formation accompanied with an up-regulation of PD-1/PD-L1 pathway (increased PD-L1 levels in tumor tissue cells and the amount of PD-1-expressing tumor-infiltrating CD8 T cells) and inhibited T cell anti-tumor function, while inhibition of PD-1/PD-L1 restored T cell anti-tumor effector function and mitigated ethanol-enhanced tumorigenesis.

## Introduction

Around 300,000 new cases of breast cancer are reported annually in the United States. Evidence suggests that alcohol consumption, even moderate drinking, is associated with increased risk of breast cancer (1). Further, epidemiological data shows that breast cancer risk does not vary by beverage type indicating that ethanol is the main causal factor (2). Given the high prevalence of alcohol drinking among women, alcohol-related breast cancer becomes a public health issue. However, the mechanism underlying ethanol-enhanced mammary tumorigenesis is unclear.

Mammary tumorigenesis is a complex disease that ought to be studied together with the microenvironment in which the tumor initiates and grows (3). The immune system is a critical component of the microenvironment and reacts against newly arising mutated cells to stop tumor formation in a process termed “immunosurveillance” (4). Tumor formation indicates a compromise of host anti-tumor immunity in which T cells play a critical role (5).

T cell anti-tumor immunity is majorly carried out by CD8 cytotoxic T cells, which attack tumor cells through their cell membrane apoptosis-inducing molecules or through the release of cytotoxic cytokines/granules (6). On the other hand, the function of CD8 T cells may be inhibited by activation of immune check point pathways, among which PD-1/PD-L1 pathway is the most studied. Activation of PD-1/PD-L1 pathway (up-regulation of PD-L1 level on tumor cells and/or increase of PD-1-expressing CD8 T cells) reduces the release of anti-tumor cytokines (e.g. IFN-γ) or cytotoxins (e.g. perforin or granzyme B) by CD8 T cells resulting in inhibition of T cell anti-tumor cytotoxic function (7).

Mishra and colleagues demonstrated that chronical ethanol exposure may up-regulate PD-1/PD-L1 expression in neuroimmune cells, thereby contributing to neurodegeneration (8). However, whether ethanol may affect PD-1/PD-L1 and how that may contribute to tumorigenesis in mammary tissue remain unclear.

FVB.Cg-Tg(Wnt1)1Hev/J female mice with activated wnt1 signaling develop spontaneous mammary tumors starting around 2-3 months of age and have been used as a mouse model for breast cancer research (9). Saboya et al. found that ethanol exposure to pregnant dams increased mammary tumor incidence in adult offspring (10). In the current study, we feed these young female mice with an ethanol-diet to mimic alcohol drinking in young women and determined the effects of ethanol on PD-L1/PD-1 pathway as well as how that affects CD8 T cell function and contributes to mammary tumorigenesis. Our data showed that ethanol exposure up-regulated PD-L1 on mammary tumor tissue cells, increased tumor-infiltrating PD-1^+^CD8^+^ T cells, inhibited cytotoxic function of CD8 T cells and enhanced mammary tumorigenesis. Importantly, these effects of ethanol exposure were inhibited by co-treatment of PD-L1 or PD-1 antibody, indicating that ethanol-enhanced mammary tumorigenesis is, at least partially, mediated by PD-1/PD-L1 signaling pathway.

## Materials and Methods

### Animals

FVB.Cg-Tg (Wnt1)1Hev/J female mice (with activated wnt1 signaling) (11) were obtained from the Jackson Laboratory and housed under a controlled temperature in the animal facility of the University of Kentucky. All experimental procedures were approved by the Institutional Animal Care and Use Committee (IACUC) of the University of Kentucky. Female mice at the age of 5 weeks were group housed (4 mice/cage) and randomly assigned into seven groups (20 mice/group): control, ethanol exposure, PD-L1 or PD-1 antibody injection, ethanol exposure plus PD-L1 or PD-1 antibody injection, and IgG isotype control group. Mice were exposed to ethanol by feed with an ethanol liquid diet (Bio-Serv, Flemington, NJ), while mice without the ethanol treatment were fed with an isocaloric liquid diet in which maltose was used to substitute for ethanol. The ethanol concentration in the ethanol diet was increased with up-titration by the following: week 1, 2% ethanol; week 2, 4% ethanol; weeks 3 and on: 6.7% ethanol. Diet was provided ad libitum for the experimental period. For ethanol-treated animals, after feed with 6.7% ethanol for one week the blood samples were collected in the early morning from the tails and the alcohol concentration was analyzed by Alcohol Analyzer AM1 (Analox Instruments, MA). For inhibition of PD-L1 or PD-1, rat anti-mouse PD-L1 antibody (rat Immunoglobulin G2b (IgG2b), Leinco Technologies) or PD-1 antibody (rat IgG2a, Leinco Technologies) were administered by intraperitoneal injection at the dose of 200 μg/mouse on the first day of the 6th, 8th and 10th week. Rat IgG2a and IgG2b (Leinco Technologies) at the same dosage were used as isotype/injection control. The treatment regimen was derived from previous studies in which PD-1 or PD-L1 antibody injection at similar dosages inhibited tumor growth with no adverse effects (12–15). The time (22 weeks of age) when 50% of the mice in control group developed mammary tumor was used to set the end point of the study so that the results can be compared to those of previous studies (16). During the experiment period, body weights of mice and general health condition were evaluated and recorded regularly. No apparent toxicity or significant body weight difference were observed among these animals. Mammary tumor size was measured using a caliper in millimeters. The mice were monitored closely after tumor formation. At the end of experiment or when tumor size reached 20 mm in maximum diameter, the mice were euthanized by Ketamine/Xylazine overdose and mammary tumors were collected, measured, and weighted. The tumor samples were thereafter used for histological and biochemical examination or flow cytometry analysis. During the experiment, a few mice (1 in ethanol, 2 in PD-L1, 1 in PD-1 and 2 in isotype control treatment groups) died for unknown reasons.

### Flow Cytometry Analysis of Single Cell Suspensions From the Mammary Tumor Tissues

The method has been published previously (15, 17, 18) with minor modification. The mammary tumor tissues for flow cytometry analysis were minced into small pieces and incubated in digestion solution containing 2 mg/ml of collagenase A (Sigma), 10 μg/ml of DNase I (Roche), and 10% FBS in Dulbecco’s PBS with MgCl_2_ and CaCl_2_ (Sigma) for 45 min with shaking at 250 rpm at 37 °C. The dissociated tumor tissues were then passed through a 100 μm cell strainer to remove the tissue debris. The cell pellets were resuspended and incubated with 5 ml 1× red blood cell lysis buffer for 4 min. The cells were then washed with 25 ml PBS by centrifuging at 350x g for 5 min, and the single cells were re-suspended in 1 ml of PBS containing 2% FCS for flow cytometry analysis. The antibodies against PD-L1, PD-1, CD45, and CD8 (all from BioLegend) were used for cell population identification. Relative isotype staining was used as negative controls. The single cells were pre-incubated with CD16/CD32 antibody (BioLegend) for 15 min and then stained with CD45 or CD8a antibody (BioLegend) to eliminate non-specific binding of Fc receptors. CD45 staining was used to gate tumor-tissue-infiltrated immune cells (CD45^+^) from non-immune cells (CD45^-^). CD8a staining was used to identify CD8 T cell population. For the detection of intracellular cytokine IFN-γ, perforin and granzyme B in CD8 T cells, a previously published protocol was used (15). Briefly, the single cells went through stimulation, fixation and permeabilization steps followed with intracellular staining with the respective antibodies (BioLegend). Acquisition of data was performed on a Cytometers BD Symphony A3 equipped with Diva software and analyzed using FlowJo 10.8 version.

### Immunohistochemistry Analysis of PD-L1^+^ Cells

The method has been published previously (15). Briefly, the mammary tumor tissues were fixed with paraformaldehyde (PFA) and embedded in paraffin. Tissue blocks were cut into 5 μm thick sections in a HM 325 rotary microtome (Thermo Scientific). Sections were then deparaffinized and placed in the antigen retrieval solution (Histo-VT One, Nacalai USA). After rinsing thoroughly in 0.1 M PBS, the sections were pre-incubated in a blocking solution and then incubated with mouse PD-L1/B7- H1 antibody (R&D systems). After that, the sections were rinsed and incubated with anti-mouse biotinylated secondary antibody IgG (H+L) (Vector Laboratories). After washing with 0.1 M PBS, the sections were incubated with Avidin/Biotin complex (Vectastain ABC Elite kit; Vector Laboratories) and then chromagen DAB (3′,3′ -diaminobenzidine, Vector Laboratories). The sections were observed under an Olympus CX43 biological microscope. For quantitative assessment of PD-L1 staining, the average optical density (OD) of the regions of interest was measured by Fiji Image J software.

### Statistical Analysis

The data were analyzed by SPSS 28 (SPSS) and bar graphs were created using GraphPad Prism 8 (Graphpad Prism). Mammary tumor free curves were generated using the Kaplan–Meier method, and the statistical significance of the difference among the different groups was measured using the Log-Rank test. Tumor size over time was analyzed by general linear model in which repeated measures were used. Univariate factorial ANOVA was used for numerical data analysis and results were presented as (mean ± SD). Partial eta squared (η^2^p) was used for calculating the effect size. The differences among groups were determined by *post-hoc* multiple comparisons. The difference between two groups was tested by independent samples *t-*test, and Cohen’s D effect size was calculated for t-test. A *p* value *<* 0.05 was considered significant.

## Results

### Ethanol Exposure Increased Mammary Tumorigenicity, Which Was Ameliorated by Co-Treatment of PD-L1 or PD-1 Antibody

**Figure 1:**
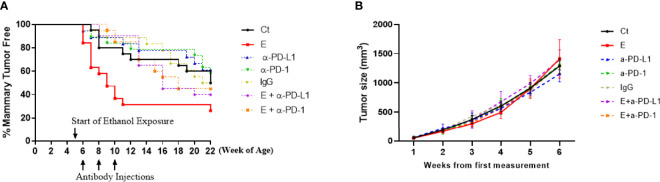
The formation of early onset mammary tumors was accelerated by ethanol exposure, which was mitigated by co-treatment of PD-L1 or PD-1 antibody. **(A)** Tumorigenesis was analyzed by Kaplan-Meier plot shown as the percentages of mammary-tumor-free animals over time up to 22 weeks of age. The formation of early onset mammary tumors was significantly accelerated by ethanol exposure (E, solid red line) compared to control group (Ct, solid black line, *p* < 0.05), which was mitigated by co-treatment of PD-L1 (E + α-PD-L1, dashed purple line) or PD-1 antibody (E + α-PD-1, dashed orange lines), *p* < 0.05. PD-L1 or PD-1 antibody treatment alone (α-PD-L1 or α-PD-1) only slightly postponed the tumor formation (*p* > 0.05). **(B)** The tumor size over time in different treatment groups was analyzed. *P* > 0.05. Per the animal welfare requirements, the mice were sacrificed before the diameter of the tumors reached 20 mm – which was usually around 6 weeks after the tumor size was first measured.

Wnt1 signaling, including both canonical and non-canonical, plays a key role not only in embryogenesis but also in the development of several cancers, such as breast cancer (19). In this study, female FVB.Cg-Tg (Wnt1)1Hev/J transgenic mice with activated wnt-1 signaling at the age of 5 weeks were randomly assigned into 7 treatment groups (20 mice/group): control, ethanol exposure, PD-L1 or PD-1 antibody injection, ethanol exposure plus PD-L1 or PD-1 antibody injection, and IgG isotype control group (see Methods for details). For ethanol-treated animals, the average blood alcohol concentration was 80.17 ± 9.06 mg/dl, a human equivalent dose of binge drinking. At the 22 weeks of age when 50% of the animals in control group developed mammary tumors, tumorigenesis was analyzed by Kaplan-Meier plot shown as the percentages of mammary-tumor-free animals over time. As shown in **Figure 1A**, ethanol exposure significantly accelerated the formation of early onset mammary tumors than that in control group (solid red line vs solid black line, *p* < 0.05), which was reversed by the injection of PD-L1 or PD-1 antibodies (purple or orange dashed line, respectively, *p* < 0.05), while PD-L1, PD-1 antibody alone or IgG treatment groups have no significant differences from control group (*p* > 0.05). Ethanol substantially shortened the time for the animals to exhibit a 50% incidence of tumors from 22 (in control group) to 9 weeks of age, whereas co-treatment of PD-L1 or PD-1 antibody postponed it from 9 to around 16 weeks. However, as shown in **Figure 1B**, ethanol exposure apparently did not alter the tumor growth after the tumors were detected.

### PD-L1 Was Up-Regulated by Ethanol Exposure on Mammary Tumor Cells

**Figure 2:**
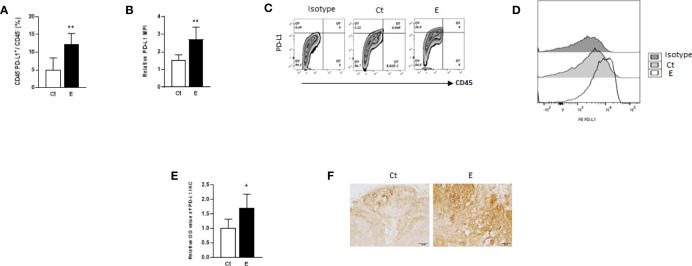
PD-L1 may be up-regulated by ethanol on the cells of mammary tumors. The percentages of PD-L1-positive tumor tissue cells (CD45^-^PD-L1^+^ cells, **(A)** and PD-L1 levels **(B)**, shown as mean fluorescence intensity, MFI, to isotype control) on the cells of mammary tumors in control (Ct) or ethanol (E)-treated groups were examined by flow cytometry. Data is presented as (mean ± SD). *N* = 5, *p < 0.05, **p < 0.01. **(C)** Representative flow cytometry plots showing the percentage of CD45^-^PD-L1^+^ cells in the mammary tumor cells with or without ethanol exposure. Isotype control plot shows non-specific staining. **(D)** Representative flow cytometry histograms showing PD-L1 levels in isotype, control and ethanol exposure group. **(E)** PD-L1 level was higher in ethanol group in comparison with control group as shown by immunohistochemical staining of PD-L1 in mammary tumor tissues from control and ethanol groups. **(F)** Representative immunohistochmistry images of PD-L1 staining of tumor tissues (scale bar: 50 µm).

The results of the animal study suggested that the PD-L1/PD-1 pathway may play an important role in ethanol-enhanced mammary tumorigenicity. Therefore, PD-L1 levels on CD45-negative cells of the tumors (most are tumor cells) between control and ethanol groups were examined by flow cytometry. As shown in **Figures 2A, C**, ethanol treatment increased the percentage of PD-L1^+^ cells in the tumors compared to that in control group. In addition, ethanol exposure also increased PD-L1 protein levels on these cells (**Figures 2B, D**). To further examine PD-L1 levels in the tumor cells, immunohistochemistry staining was performed to the mammary tumor tissue sections. As shown in **Figures 2E, F**, PD-L1 expression was higher in the tumor tissues of ethanol treated group than that of control group.

### Ethanol Exposure Increased PD-1^+^CD8^+^ T Cells in the Mammary Tumor Microenvironment

**Figure 3:**
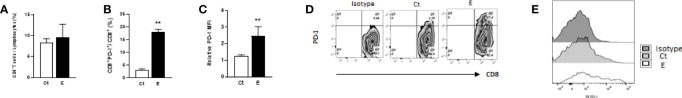
Ethanol exposure increased PD-1^+^CD8^+^ T cells in the mammary tumor tissues. Ethanol exposure did not alter the proportion of tumor-infiltrating CD8 T cells **(A)** but increased the percentage of PD-1^+^CD8^+^ T cells **(B)** and PD-1 levels on CD8 T cells **(C)** in the mammary tumor tissues. Data is presented as (mean ± SD). *N* = 5, **p < 0.01. **(D)** Representative flow cytometry plots showing the percentages of PD-L1^+^CD8^+^ T cells in the mammary tumor tissues with or without ethanol exposure. **(E)** Representative flow cytometry histograms showing PD-1 levels on tumor-infiltrating CD8 T cells in the isotype, control and ethanol-exposed animals.

Since the immune cells in the tumor microenvironment may promote or inhibit tumor growth (20) and the host anti-tumor immunity is mainly carried out by CD8 T cells, we next examined the effects of long-term ethanol exposure on tumor-infiltrating CD8 T cells. The flow cytometry data indicated that ethanol exposure did not alter the proportion of these T cells significantly (**Figure 3A**). In addition, since the PD-1/PD-L1 pathway could be used by tumors to escape immune surveillance, we also examined the effects of ethanol exposure on the percentage of PD-1^+^CD8^+^ T cells as well as on the level of PD-1 on tumor-infiltrating CD8 T cells. As shown in **Figures 3B–E**, ethanol exposure significantly increased the percentage of PD-1^+^CD8^+^ T cells (**Figures 3B, D**) as well as PD-1 protein levels on these cells (**Figures 3C, E**).

### The Cytotoxic Function of Tumor-Infiltrating CD8 T Cells Was Compromised by Ethanol Exposure, Which Was Restored by the Co-Treatment of PD-L1 or PD-1 Antibody

**Figure 4:**
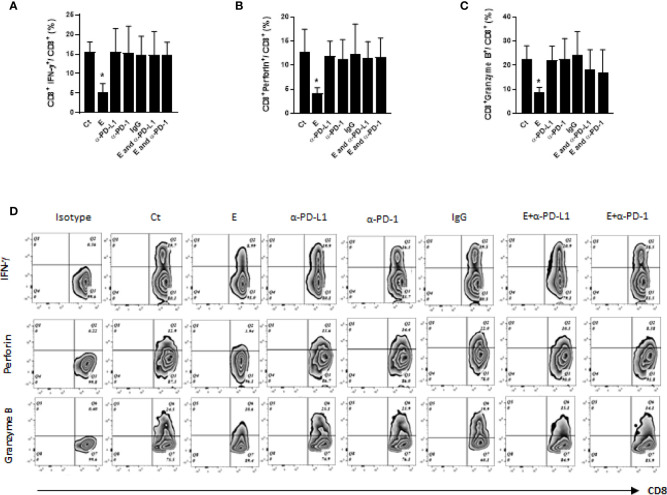
The cytotoxic function of CD8 T cells was compromised by ethanol exposure and was restored by co-treatment of PD-L1 or PD-1 antibody. **(A-C)** The percentages of IFN-γ^+^CD8^+^, perforin^+^ CD8^+^ and granzyme B^+^CD8^+^ T cells in tumor tissues were compared among different treatment groups. **(D)**. Representative flow cytometry plots for **(A–C)**. Data is presented as (mean ± SD). *N* = 5. *p < 0.05.

CD8 cytotoxic T cells play a vital role in anti-tumor immunity. The tumor-infiltrating CD8 T cells kill the tumor cells by releasing apoptosis-inducing cytotoxic proteins (e.g. interferon gamma, IFN- γ) or granules (e.g. perforin and granzyme B) (21). Therefore, the effects of ethanol exposure on cytotoxic function of CD8 T cells were first examined by comparing the proportion of IFN-γ^+^CD8^+^ T cells in the mammary tumor tissues among different groups. Our data showed the percentage of IFN-γ-producing CD8 T cells were significantly decreased in the mammary tumor tissues of ethanol-exposed mice, which was restored by the co-treatment of PD-L1 or PD-1 antibody (**Figure 4A**). Next, we analyzed the effects of ethanol exposure on CD8 T cells producing perforin or granzyme B. As shown in **Figures 4B, C**, the percentages of perforin^+^CD8^+^ and granzyme B^+^CD8^+^ T cells were reduced by ethanol exposure, which were also ameliorated by the co-treatment of PD-L1 or PD-1 antibody.

## Discussion

Ethanol may promote breast cancer through multiple mechanisms, such as enhancing cancer onset, progression, and aggressiveness (22–24). Accumulating evidence suggests that compromised host anti-tumor immunity may also play an important role in ethanol-promoted breast cancer. Recent clinical practice has highlighted T cell anti-tumor immunity regulated by immune checkpoint pathways in cancer development and treatment. Unlike most of the studies that have been focused mostly on the treatment effects of PD-1/PD-L1 inhibitors on existing tumors, the current study explored a possible role of PD-1/PD-L1 in ethanol-enhanced mammary tumorigenesis.

In the study, the transgenic mice were exposed to ethanol from 5 to 22 weeks of age achieving a blood alcohol concentration around 80 mg/dl. It mimicked a binge alcohol drinking pattern in young women (25) since that is the drinking pattern and window of time that make breast tissue most susceptible to alcohol as a carcinogen (26). Our data showed that this ethanol exposure accelerated the formation of early onset mammary tumors in the mice. Ethanol substantially shortened the time from 22 (in control group) to 9 weeks of age for 50% of the animals to exhibit mammary tumors (**Figure 1A**). Interestingly, ethanol exposure did not alter tumor growth kinetics (**Figure 1B**), suggesting a more specific effect of ethanol on mammary tumor initiation than growth. Our data, thus, shows for the first time how ethanol enhances mammary tumorigenesis in animals. Importantly, inhibition of PD-1/PD-L1 pathway by injection of PD-1 or PD-L1 antibodies reversed the trend and extended the age with 50% incidence from 9 to around 16 weeks, indicating the importance of the PD-1/PD-L1 immune check point pathway in ethanol-promoted mammary tumorigenesis. The effects of ethanol may be more specifically associated with PD-1/PD-L1 pathway since the animals with antibody injection alone did not show significant differences in tumor initiation/growth compared to the control group. The method of antibody injection was modified from previous studies that showed an inhibition of tumor growth with no adverse effects (12, 14, 15). In addition, the antibody administration was performed at the early stage of tumor formation since the goal was to study the mechanism underlying ethanol-promoted mammary tumorigenesis and we wanted to avoid possible complications caused by the treatment effects of the antibodies on existing tumors. Of note, Wnt-1 signaling may not directly contribute to ethanol’s action in enhancing mammary tumorigenesis as it is already activated in this mouse strain (11).

CD8 cytotoxic T cells are the main immune cell population to carry out host anti-tumor immunity by killing the tumor cells *via* releasing their apoptosis-inducing molecules (e.g. IFN-γ) or cytotoxic granules (e.g. perforin or granzymes). It has been shown that alcohol abuse may adversely affect T cell function (27), chronic alcohol exposure promotes systemic pro-inflammatory IFN-γ and IL-17 responses in mice (28), and alcohol consumption affect the immune phenotype of CD8 T cells (29) as well as the response of CD8 T cells against virus infection (30). In the current study our data confirmed the immunotoxic effects of ethanol on T cell function by showing that long-term ethanol exposure inhibited the cytotoxic effector function of CD8 T cells (with reduced CD8 T cells that produce IFN-γ, perforin or granzyme B molecules, **Figure 4**). More importantly, the effects of ethanol on T cell function and tumorigenesis can be mitigated by co-treatment of PD-1 or PD-L1 antibodies, suggesting ethanol-up-regulated PD-1/PD-L1 inhibited T cell cytotoxicity, which contributed to ethanol-enhanced mammary tumorigenesis. The results may shed a light on the preventive strategies against mammary tumorigenicity promoted by alcohol consumption. Currently, the mechanism underlying ethanol-enhanced PD-1/PD-L1 signaling is under investigation.

## Data Availability Statement

The original contributions presented in the study are included in the article/supplementary material. Further inquiries can be directed to the corresponding authors.

## Ethics Statement

The animal study was reviewed and approved by the Institutional Animal Care and Use Committee (IACUC) of the University of Kentucky.

## Author Contributions

WX, data curation, methodology, investigation, formal analysis, and writing-original draft. LW, methodology and investigation. MX, data curation, investigation, and formal analysis. JL, conceptualization and funding acquisition. GC, conceptualization, supervision, funding acquisition, writing-review, and editing. All authors contributed to the article and approved the submitted version.

## Funding

This work was supported by NIH grants AA017226 to JL, AA026787 to GC and P30CA177558 to the Shared Resource Facilities of the University of Kentucky Markey Cancer Center.

## Conflict of Interest

The authors declare that the research was conducted in the absence of any commercial or financial relationships that could be construed as a potential conflict of interest.

## Publisher’s Note

All claims expressed in this article are solely those of the authors and do not necessarily represent those of their affiliated organizations, or those of the publisher, the editors and the reviewers. Any product that may be evaluated in this article, or claim that may be made by its manufacturer, is not guaranteed or endorsed by the publisher.
